# Changes in the Brain in Temporal Lobe Epilepsy with Unilateral Hippocampal Sclerosis: An Initial Case Series

**DOI:** 10.3390/healthcare10091648

**Published:** 2022-08-29

**Authors:** Sung Chul Lim, Juhee Oh, Bo Young Hong, Seong Hoon Lim

**Affiliations:** 1 Department of Neurology, St. Vincent’s Hospital, College of Medicine, The Catholic University of Korea, Seoul 06591, Korea; 2 Department of Rehabilitation Medicine, St. Vincent’s Hospital, College of Medicine, The Catholic University of Korea, Seoul 06591, Korea

**Keywords:** temporal lobe epilepsy, hippocampal sclerosis, TLE, HS, epilepsy, seizure

## Abstract

Temporal lobe epilepsy (TLE) is a network disorder of the brain. Network disorders predominately involve dysregulation of hippocampal function caused by neuronal hyperexcitability. However, the relationship between the macro- and microscopic changes in specific brain regions is uncertain. In this study, the pattern of brain atrophy in patients with TLE and hippocampal sclerosis (HS) was investigated using volumetry, and microscopic changes in specific lesions were observed to examine the anatomical correspondence with specific target lesions using diffusion tensor imaging (DTI) with statistical parametric mapping (SPM). This retrospective cross-sectional study enrolled 17 patients with TLE and HS. We manually measured the volumes of the hippocampus (HC), amygdala (AMG), entorhinal cortex, fornix, and thalamus (TH) bilaterally. The mean diffusivity and fractional anisotropy of each patient were then quantified and analyzed by a voxel-based statistical correlation method using SPM8. In right TLE with HS, there was no evidence of any abnormal diffusion properties associated with the volume reduction in specific brain regions. In left TLE with HS, there were significant changes in the volumes of the AMG, HC, and TH. Despite the small sample size, these differences in conditions were considered meaningful. Chronic left TLE with HS might cause structural changes in the AMG, HC, and TH, unlike right TLE with HS.

## 1. Introduction

The temporal lobe is the most epileptogenic part of the human brain. Temporal lobe epilepsy (TLE) is a network disorder of the brain that predominately involves dysregulation of hippocampal function caused by neuronal hyperexcitability [1,2,3]. Chronic seizure activity may alter the diffusion properties of a seizure network. Recently, changes in the white matter network topology and structural connectivity were found in patients with left nonlesional TLE [4,5].

The seizure network is larger than the seizure onset zone. The Papez circuit has especially strong anatomical connections to the medial temporal area and modulates TLE [6,7]. Hippocampal sclerosis (HS) is a key component in the generation and propagation of seizures in TLE [8,9]. Regional atrophy of specific brain regions, especially the hippocampus (HC) or temporal lobe, is common in epilepsy [9,10]. In addition, the amygdala (AMG) and the mean diffusivity (MD) and fractional anisotropy (FA) of the thalamus (TH) are increased bilaterally in patients with TLE [8,11]. However, the relationship between the macro- and microscopic changes in specific brain regions is not clear.

The correlations of morphological and structural changes with clinical information are very important for patients with TLE and their clinicians. We hypothesized that the morphology and structure of the seizure network change with chronic seizures in patients with TLE. Therefore, in this study, we investigated the pattern of brain atrophy in patients with TLE and HS using volumetry and observed regional microscopic changes in specific lesions that correspond anatomically with the specific target lesions, with small case series restricting the bias and uncovering more clinically meaningful insight.

## 2. Methods

### 2.1. Study Design and Participants

This retrospective cross-sectional study enrolled 17 subjects with TLE and unilateral HS recruited from the Department of Neurology, St. Vincent’s Hospital, between January 2013 and December 2017. All subjects had (1) TLE diagnosed by a neurologist specializing in epilepsy based on video EEG monitoring [12] and (2) unilateral HS confirmed by MRI. Exclusion criteria were (1) any other brain parenchymal disease, (2) any other neurovascular disease, or (3) surgery for TLE. We enrolled all consecutive subjects according to the inclusion and exclusion criteria for diminishing selection bias. This study was a retrospective observational study for TLE and unilateral HS; we did not calculate the exact sample size and enrolled consecutive subjects suitable for criteria within five years. In addition, we tried to estimate the appropriate number of samples based on previous studies, which varied from 12 to 24 [5,13]. We decided the sample size should be over twelve subjects.

The study protocol was reviewed and approved by the Institutional Review Board of Catholic University, College of Medicine (Registry No. VC18RESI0215); the need for informed consent was waived by the board.

### 2.2. DTI Acquisition and Regional Volumetry

DTI was performed using a 3.0-T MRI scanner (MAGNETOM^®^ Verio, Siemens, Erlangen, Germany) equipped with a six-channel head coil. Data were acquired as single-shot spin-echo echo-planar images, with axial slices covering the entire brain across 76 interleaved (2.0 mm thick) slices (no gap), using the following parameters: repetition time/echo time, 14,300/84 ms; field of view, 224 × 224 mm^2^; matrix, 224 × 224; voxel size, 1 × 1 × 2 mm^3^; number of excitations, 1. Diffusion sensitizing gradients were applied in 64 noncollinear directions with a b-value of 1000 ms/mm^2^ [14]. The b0 images were scanned before acquiring the diffusion-weighted images, with 65 volumes total [15,16,17]. Raw diffusion-weighted data were corrected for geometric distortion secondary to eddy currents using a registration technique based on a geometric model of distortion, as described by Lehéricy et al. [18,19]. The FA and MD were mapped using homemade software based on MATLAB 7.4 (The MathWorks, Natick, MA, USA).

The raw DTI data were transferred to a personal computer, equipped with the freely shared programs Volume-One (ver. 1.56) and dTV (ver. II). In addition to the b0 and b1000 images (isotropic DWI), diffusion tensor maps of the FA, apparent diffusion coefficient, and color-coded images were generated. The diffusion tensor parameters were calculated on a voxel-by-voxel basis. Then, the diffusion eigenvectors and corresponding eigenvalues (x1, x2, x3) were acquired. The eigenvector (e1) associated with the largest eigenvalue (x1) was assumed to represent the local fiber direction [20].

Anatomical regions of interest on T1-weighted images were obtained using the segmentation tool FIRST 1.1 integrated within the FSL software. We performed volumetry of the HC, AMG, entorhinal cortex (EC), fornix (FX), and TH in each subject and each hemisphere. The medical image analysis program, Analyze 7.5 (Mayo Foundation, Rochester, MN, USA), was used to measure the volumes [21]. The anatomical boundaries of the HC were measured from front (the alveus) to back (the point where the hippocampal tail disappears); the fimbria was excluded [21]. Before the statistical analysis, individual volumes were corrected using a multiplicative scaling factor derived from affine transformation calculated using the SIENAX tool in the FSL software. This scaling factor was computed as the determinant of the affine transform connecting each individual to the standard Montreal Neurological Institute template [22].

### 2.3. SPM Data Analysis

For voxel-based analysis, data were analyzed using SPM8 (Wellcome Department of Cognitive Neurology, http://www.fil.ion.ucl.ac.uk, accessed on 2 December 2018) running on MATLAB 7.4. Nondiffusion-weighted images (B0) from each subject were normalized to a mean T2 template in the SPM8 package using linear steps with 12 degrees of freedom and a 7 × 8 × 7 nonlinear warp; these deformation parameters were applied to the MD and FA images from each subject. The normalized data were then smoothed using an 8 mm full-width-at-half-maximum isotropic Gaussian kernel. Statistical analysis was performed on all patients as a single group. The resulting maps were thresholded at a *p*-value of <0.05 using family-wise error to correct for multiple comparisons (random field theory) [23]. We also present the coordinates and corresponding regions in Talairach space, derived using the algorithm presented at http://www.mrc.cbu.cam.ac.uk/Imaging/Common/mnispace (accessed on 2 December 2018) [24].

## 3. Results

**Figure 1:**
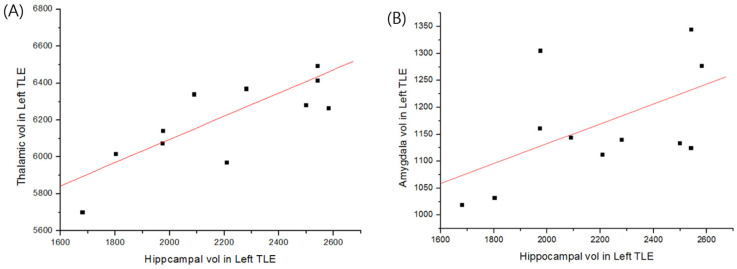
The relationships between the volumes (mm^3^) of the amygdala, hippocampus, and thalamus in left temporal lobe epilepsy with hippocampal sclerosis (left TLE with HS). (**A**) The volume of the left hippocampus was positively correlated with that of the left thalamus in patients with left TLE (*p* = 0.00688). (**B**) No correlation was observed between the left hippocampus and left amygdala (*p* = 0.06832), entorhinal cortex, or fornix (not shown) in the patients with left TLE.

**Figure 2:**
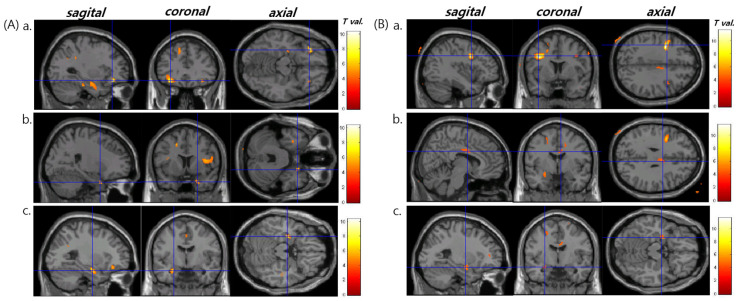
Statistical parametric mapping (SPM) of the fractional anisotropy (FA) changes in left temporal lobe epilepsy with hippocampal sclerosis (left TLE with HS). (**A**) Reduced FA correlated with decreased hippocampal volume: (**a**) bilateral inferior frontal areas; (**b**) right hippocampus; (**c**) left amygdala and hippocampus. (**B**) Reduced FA correlated with decreased thalamic volume: (**a**) left prefrontal area; (**b**) right cingulate gyrus; (**c**) left amygdala (corrected for family-wise error, *p* < 0.05).

Table 1 summarizes the subjects’ demographic and clinical characteristics. We did not identify any brain area with abnormal diffusion properties associated with a volume reduction in each region of interest in patients with right TLE and HS. However, in the patients with left TLE and HS, there was a correlation between the changes in HC and TH volumes (Figure 1A) but not the AMG, EC, or FX volumes (Figure 1B). The volumes of the hippocampus, thalamus, and amygdala in all subjects are presented in Table 2. In the patients with left TLE and HS, the decreases in FA in the inferior frontal lobe bilaterally, HC bilaterally, and left AMG correlated with a decreased hippocampal volume (Figure 2A, Table 3). The brain regions exhibited diffusion abnormalities in the left or right TLE with HS. In the same patients, decreases in FA in the left prefrontal lobe, right cingulate gyrus, and left AMG correlated with decreased TH volume (Figure 2B, Table 3).

## 4. Discussion

We hypothesized that chronic seizures would alter the morphology and structure of the seizure network in patients with TLE. We detected a reduced FA and volume in the AMG, HC, and TH in patients with left TLE and HS but not those with right TLE and HS in small case series. These findings are consistent with reports of structural and pathological differences between right and left TLE [4,13,25,26]. In addition, compared to the previous normative volume of HC (2411 mm^3^), the size of the hippocampus in patients with long-standing TLE was atrophied [27]. Our results present supportive evidence that left TLE with HS may have a distorted network among HS, AMG, and TH; in contrast, right TLE with HS might be a distortion of HC itself. In addition, based on our results, the alteration of white matter may be related to the pathomechanism and adaptive progress of TLE itself for preserving critical cognitive functions and may have a predictive value for differentiation of left TLE from right TLE, potentially increasing the importance of diffusion imaging in clinical practice. The results also suggest that the abnormalities in patients with focal TLE are not necessarily restricted to the temporal lobes but might extend to other brain regions. The previous study was consistent with our results [25]. However, in the treatment of temporal lobe epilepsy research, these recent findings did not immerse clinically [28]. Thus, we present novel findings for differences between right TLE and left TLE. Based on our results and a previous study [25,28]. The treatment will have to be approached differently; focal surgery may be beneficial for chronic left TLE with unilateral HS than chronic right TLE with unilateral HS.

A recent fMRI study showed the dynamic network abnormalities of TLE with increased network integration between the hippocampal and frontoparietal lobes [29]. Another study demonstrated that left TLE implied altered brain structural connectivity with the contralateral mesial temporal lobe in contrast to right TLE [30]. In addition, the left TLE exhibits a more severe alteration of white matter and networks in the brain [4,31]. These findings are also supportive of our results. Furthermore, these findings may be useful for surgery for TLE, recently suggested by a new prediction model [32]. Combining these findings with our results, understanding the different pathomechanisms for right and left TLE would be useful for clinicians and may provide a therapeutically valuable basis.

We did not identify changes in DTI parameters or the regional volume of the EC or FX in either TLE group. The FX is often used for deep brain stimulation to control TLE [6]. However, no structural changes were found in either of our TLE groups. The EC has an important role in the TLE network [33,34]. Our negative findings are consistent with a neuropathological study that found no significant difference in mean neuron densities in the EC region between HS and left TLE groups or postmortem controls [35]. Thus, the EC and FX may play a role in the functional network but not the structural network in patients with TLE. However, interpreting these results could be limited based on the small sample size.

In our investigation of TLE with HS, we used several methods to overcome bias. First, our inclusion was narrow and homogenous, recruiting only patients with TLE over ten years without surgery and excluding patients with a history of invasive treatments or surgery and enrolled in a consecutive manner. However, the small number of samples was a significant limitation for our study. To answer the remaining questions and solve equivocal results, further research is needed to address the remaining questions with a large cohort study.

In conclusion, chronic left TLE with unilateral HS might cause structural changes in the AMG, HC, and TH, in contrast to right TLE with unilateral HS.

## Figures and Tables

**Table 1:** Patient demographics and clinical information.

Subject	Age	Sex	Side	HS	Onset (Year)	Duration (Year)	Frequency of CPS (Number/Month)	Frequency of GTCS (>10, Total)
1	28	F	Left	Yes	8	20	15	No
2	37	F	Left	Yes	23	14	3	Yes
3	41	F	Left	Yes	29	12	11	No
4	44	F	Left	Yes	21	23	2.5	Yes
5	41	M	Left	Yes	6	35	5	No
6	28	F	Right	Yes	16	12	2	No
7	41	F	Right	Yes	19	23	4	Yes
8	22	M	Left	Yes	9	13	4	No
9	28	F	Left	Yes	17	11	1	No
10	30	M	Left	Yes	19	11	2	Yes
11	48	F	Left	Yes	19	29	4	No
12	43	F	Left	Yes	31	12	3	No
13	35	F	Left	Yes	9	26	0.7	Yes
14	32	M	Right	Yes	11	21	4	No
15	43	F	Right	Yes	24	19	2	Yes
16	38	F	Right	Yes	26	12	1.5	Yes
17	46	M	Right	Yes	33	13	3	No

**Table 2:** Volume of the hippocampus, thalamus, and amygdala in TLE with HS.

Subject	Side	Hippocampus (mm^3^)	Thalamus (mm^3^)	Amygdala (mm^3^)
1	Left	2291	6382	1132
2	Left	1978	6075	1168
3	Left	1990	6120	1309
4	Left	2579	6225	1269
5	Left	1677	5702	1022
6	Right	1874	5903	1125
7	Right	2460	6200	1190
8	Left	2093	6318	1138
9	Left	1808	6098	1030
10	Left	2511	6281	1125
11	Left	2521	6402	1119
12	Left	2528	6481	1341
13	Left	2212	5931	1113
14	Right	2373	5980	1215
15	Right	2295	6112	1341
16	Right	2334	5983	1176
17	Right	1924	5723	1095

**Table 3:** Brain regions exhibiting diffusion abnormalities in the left or right TLE with HS.

		Side	Region	Cluster-Level ^k^E	Peak-Level		
					T-Score	Z-Score	Coordinates (mm, mm, mm)
Left TLE-HS	Reduced FA correlated with decreased hippocampal volume	ipsi	Inferior frontal lobule	568	11.92	5.12	−2 −70 14
		contra	Inferior frontal lobule	155	6.62	4.02	−4 −74 −16
		ipsi	Hippocampus	112	10.4	4.87	−30 34 −14
		contra	Hippocampus	32	7.49	4.26	42 0 26
		ipsi	Amygdala	306	7.04	4.13	4 2 2
	Reduced FA correlate with decreased thalamic volume	ipsi	Prefrontal lobule	165	8.3	4.45	−12 −60 34
		contra	Cingulate gyrus	261	11.73	5.09	−36 6 34
		ipsi	Amygdala	49	6.44	3.96	−16 14 66
Right TLE-HS	Reduced FA correlate with decreased hippocampal volume	ipsi and contra	None				
	Reduced FA correlate with decreased thalamic volume	ipsi and contra	None				

## Data Availability

The data presented in this study are available upon request from the corresponding author.
